# Ohio State Dental Society

**Published:** 1876-08

**Authors:** J. M. Porter


### OHIO STATE DENTAL SOCIETY.

BY J. M. PORTER, D. D. S.

When secretary of the Ohio State Dental Society, in 1874 and 1875, I noticed that a number of members failed to pay their annual dues, and I also noticed that the class who failed to pay their dues were rapidly increasing. To illustrate this fact, I will give the number who failed to pay their dues, for five years: In 1871, nine failed to pay; in 1872, twenty-two failed to pay; in 1873, thirty-nine failed to pay; in 1874, sixty failed to pay; in 1875, *seventy-four* failed to pay their annual dues. There are now on the roll book, one hundred and twenty-four names. These figures show that in 1875, nearly *two-thirds* of the Ohio State Dental Society failed to pay their annual dues. It is a fact well known to the members of this Society, that two assessments of ten dollars each have been made. At the time these assessments were made, there were one hundred and one names on the roll book. Of this number, twenty have paid twenty dollars each; eleven have paid ten dollars each, and two have paid five dollars each. Of the one hundred and one names mentioned as being liable, seventy-nine are liable for *both* assessments, and twenty-two are only liable for the last assessment.

Now if all of these members had paid their assessments, they would have put into the hands of the Treasurer, one thousand and eight hundred dollars. Instead of getting this amount, the Treasurer has only succeeded in collecting five hundred and twenty dollars, and he has had to write and beg of men to pay these assessments, *after they voted to make them.* In the matter of these assessments, the figures prove a deficiency of twelve hundred and eighty dollars. The deficiency in annual dues in the past five years, amounts to six hundred and twelve dollars. Add to this amount the amount of assessments unpaid, and we have the modest sum of eighteen hundred and ninety-two dollars.

I have presented these figures for the purpose of showing why our Society is in debt.

Those who were present when these assessments were made, will remember that the resolutions to make the assessments were passed unanimously. I do not know the amount of our indebtedness, but I am quite sure we would be free from debt if every man had met his obligations like a man ought to, especially after voting to instruct the committee to act as they did. At the annual meeting of the State Society in 1872, the following resolution was offered by the writer, and passed:

*Resolved,* That all members of this Society, who shall neglect to pay their dues for two consecutive years, shall not be permitted to resume their membership until first paying all back dues.

If I were offering that resolution again, I should change it so as to prevent their ever resuming their membership after they had failed to pay their dues for two years.

I can not see the advantage in having a class of members in the Ohio State Dental Society, who not only refuse to do any thing, but who also refuse to pay anything in support of the Society.

And now what shall the remedy be? I am in favor of adopting the plan suggested to me in a recent letter from a member of the Society. He said: " At our next meeting the roll must be purged, and all who refuse to pay up their dues and assessments be stricken from the list. It is folly to retain men who refuse to do their duty."

I leave this subject and these figures to the consideration of the members of the Society, trusting that some plan may be adopted, which will enable the good work of the Society to be carried on in the future as it has been in the past.

